# Healthcare professionals consensus on purpose, content and frequency of physical activity for older adults during the non-weight-bearing rehabilitation: an iterative consensus study

**DOI:** 10.1007/s41999-026-01463-5

**Published:** 2026-04-12

**Authors:** Elma van Garderen, Mandy Visser, Wilco P. Achterberg

**Affiliations:** 1 Department of Public Health and Primary Care, Leiden University Medical Center, Hippocratespad 21, 2333 ZD Leiden, The Netherlands; 2 Topaz, Aaltje Noordewierlaan 50, 2324 KS Leiden, The Netherlands

**Keywords:** Delphi study, Consensus, Non-weight-bearing, Healthcare professionals, Geriatric, Rehabilitation, Physical activity

## Abstract

**Background:**

Among older adults who are temporarily restricted from weight-bearing on a lower extremity, inactivity is prevalent, and there is limited evidence-based guidance available regarding physical activity programs for older adults with such restrictions. However, healthcare professionals do have ample experience working with those patients. This study explored their experiences and aimed to reach consensus among healthcare professionals on the purpose, desired content, and frequency of physical activity for older adults during non-weight-bearing rehabilitation.

**Methods:**

A three-phase iterative design was followed. Phase one consisted of focus group discussions with healthcare professionals. In phase two, panel discussions were used to extract relevant information from phase one. The relevant information was used as statements in phase three, a Delphi consensus study aimed to reach consensus among healthcare professionals.

**Results:**

In total, 76 healthcare professionals participated in the first Delphi round. 47 consensus statements, based on the first two phases, were grouped under eight themes derived from the focus group and panel discussions. Consensus was reached on 38 of 47 statements in round one. In round two, 51 participants reviewed eight revised or new statements, of which six reached consensus. The final two statements that did not reach consensus were revised based on the participants’ comments and expert feedback.

**Conclusion:**

This study provides a consensus-based foundation for developing physical activity interventions for older adults during non-weight-bearing rehabilitation. Although there was broad agreement on most statements, participants emphasized the importance of tailoring interventions to each patient’s individual condition, abilities, and goals.

**Supplementary Information:**

The online version contains supplementary material available at 10.1007/s41999-026-01463-5.

## Key summary points

**Aim:**

We aimed to achieve consensus among healthcare professionals on the purpose, content, and frequency of physical activity for older adults during non-weight-bearing rehabilitation.

**Findings:**

Consensus was reached on 44 statements across eight themes: definition, purpose, frequency and content of physical activity, informing patients and informal caregivers, involving informal caregivers, stimulating and motivating physical activity, and the role of the healthcare professional.

**Message:**

- Physical activity should be tailored to the patient’s individual condition, abilities, and goals.
- Physical activity should aim to minimize physical deconditioning and restore trust in one’s body, combining scheduled therapy with self-regulated exercises.
- All healthcare professionals play a role and should possess basic knowledge of physical activity and recovery.

**Supplementary Information:**

The online version contains supplementary material available at 10.1007/s41999-026-01463-5.

## Introduction

Hospitalized older adults recovering from illness and injuries spend most of their waking hours lying down or sitting [1]. This sedentary behavior continues during inpatient geriatric rehabilitation, with an average walking time of 37 min at day 15 [2]. Even three months after surgery, sedentary behavior remains highly prevalent: older adults recovering from a hip fracture spend an average of 11 h per day in prolonged sedentary behavior [3]. Physical inactivity is associated with various negative physical and mental health outcomes, including increased mortality, sarcopenia, functional limitations and depression [4]. Therefore, promoting early physical activity during hospitalization and inpatient geriatric rehabilitation may help prevent these adverse health outcomes [5].

According to healthcare professionals, one of the barriers to physical activity among patients aged 65 or older is the restriction of weight-bearing on a lower extremity [6]. They view these restrictions as an obstacle to strength training, noting that the use of training equipment often conflicts with the limitations imposed by non-weight-bearing (NWB) protocols [6]. Healthcare professionals agree that a personalized physical activity program should be developed at the onset of a period with weight-bearing restrictions [7]. Such a program may help to overcome this barrier. However, what this program should entail and how to effectively encourage older adults with weight-bearing restrictions to engage in physical activity remain unclear [8].

There is limited evidence-based guidance available regarding physical activity programs for older adults with weight-bearing restrictions. A previous study explored the perspectives of NWB patients and their informal caregivers on the content and frequency of physical activities, as well as the motivational factors that facilitate or hinder engagement [9]. However, since healthcare professionals have extensive experience working with NWB patients, it is important to gain insight into their perspectives as well. This study explores their views on physical activity during the NWB period and aims to reach consensus on these views. Our research question was: According to the physiotherapist, occupational therapist, nurse and the orthopedic and trauma surgeons, what is the purpose, desired content and recommended frequency of physical activity for NWB patients?

## Methods

### Design

This iterative consensus study, conducted between May 2024 and July 2025, was structured into three phases. The first phase involved focus group discussions with healthcare professionals to gather insights on physical activity for older adults during the NWB period. In phase two, relevant information from phase one—supplemented by findings from previous studies—was selected for inclusion in the consensus study. This selection was carried out in collaboration with the expert panel, peer group, and research team. The third phase consisted of a consensus study among healthcare professionals using the Delphi method [10]. The Medical Ethics Committee of the Leiden University Medical Center reviewed the protocol and concluded that the study is not subject to the Medical Research Involving Human Subjects Act (WMO) (Protocol number 23-3125).

### 1) Phase one, focus group discussions

Phase one consisted of two semi-structured focus group discussions conducted in May and June 2024, using an inductive qualitative approach [11]. The focus groups were designed to encourage open dialog and stimulate ideas regarding physical activity during the NWB period. The objective was to gather a broad range of perspectives and insights on the desired content, frequency, and purpose of physical activity during the NWB period, serving as a foundation for the subsequent consensus study.

#### Participant population and recruitment

The focus group discussions were held in two geriatric rehabilitation centers (GR centers) and were scheduled to last up to 90 min. Physiotherapists, occupational therapists, and nurses were invited to participate. The aim was to include five participants in each focus group, with at least one representative from each profession. Healthcare professionals were eligible to participate if they worked in GR centers that admit, among others, NWB patients and had experience working with this population. Additional inclusion criteria included an adequate level of Dutch language comprehension and expression, and the absence of any conflicts of interest that could influence their input. Eligible participants were included after signing an informed consent form, following receipt of written information about the study.

#### Data collection

The focus group discussions were audio-recorded. When the group size exceeded five participants, an additional researcher with prior knowledge of the topic was present to take notes, ensuring that all significant contributions were accurately captured.

#### Data analysis

The recorded discussions were transcribed and thematically analyzed using the framework method [11]. The program Atlas.ti was used for coding the data and Excel was used for charting the data. After familiarization with the data, the transcripts were openly coded, leading to the identification of sub-themes and main themes. In this context, interpreting the data involved formulating statements for the consensus study, with each sub-theme serving as the basis for a statement. This process was carried out by one researcher and reviewed by the research team to ensure that no information was overlooked or misinterpreted.

### 2) Phase 2, panel discussions

Before initiating the panel discussions, data from the focus group sessions—supplemented by findings from previous studies [9, 12]—were formulated into statements by the researcher. This process was reviewed by the research team to ensure accuracy and completeness.

#### Participant population

The expert panel consisted of eight researchers with healthcare backgrounds as (para-)medical professionals. The research team included WA, MV, and EvG, and the peer group comprised representatives of patients and their informal caregivers.

#### Data collection and analysis

Data were collected using the Castor EDC platform [13]. The expert panel and research team assessed the relevance of each statement using a 5-point Likert scale (0 = irrelevant, 4 = relevant) for inclusion in the consensus study. Participants were also invited to provide comments and suggest additional statements for each theme. Descriptive statistical analysis was performed, and all comments were documented. Following this assessment, an online meeting was held to discuss the outcomes and to establish criteria for excluding statements. These criteria were subsequently presented to the peer group and revised when necessary.

### 3) Phase 3, consensus study

Phase three aimed to establish consensus among healthcare professionals regarding the desired content, frequency, and purpose of physical activity for the NWB patient during the NWB period, using the Delphi method. This iterative process involved multiple rounds of questionnaires, with feedback from each round incorporated into the next [10]. Subsequent rounds included statements that had not yet reached consensus, along with the results and comments from the previous round, as well as newly suggested statements from the participants. Between rounds, the expert panel and research team were consulted to determine which statements should be included or reformulated for the subsequent questionnaire. This process continued until all statements reached consensus or, based on the feedback, were deemed unsuitable to proceed, with a maximum of three rounds.

#### Participant population and recruitment

A convenience sampling method was used, involving the University Network of the Care sector South Holland, a geriatric rehabilitation symposium, and the research teams’ networks with rehabilitation centers and hospitals. Information was provided verbally or via an information letter. Potential participants from GR centers admitting NWB patients included physiotherapists, occupational therapists, and nurses. To be eligible, participants were required to have at least one year of experience working with NWB patients, an adequate level of Dutch language comprehension and expression, and no conflicts of interest that could influence their input. The goal was to recruit 20 physiotherapists, 20 occupational therapists and 20 nurses for the first round, accounting for an anticipated 20% dropout rate between rounds. This would ensure that at least 10 professionals from each discipline remained for the final round.

Eligible participants from hospitals were orthopedic and trauma surgeons with at least one year of experience advising older adults to follow NWB protocols. They also needed an adequate level of Dutch language comprehension and expression, and the absence of any conflicts of interest that could influence their input. The goal was to recruit 20 surgeons, accounting for an expected 20% dropout rate between rounds, to ensure that at least 10 surgeons remained for the final round.

#### Data collection/questionnaire rounds

Participants were invited to complete an online questionnaire via the Castor EDC platform. Each questionnaire was estimated to take no more than 30 min to complete [14].

Round One: the first round began with informed consent and background questions, including profession, age, years of working experience, and workplace (hospital or GR center), followed by the consensus statements. Participants rated each statement on a 5-point Likert scale (1 = strongly agree, 5 = strongly disagree) and were given the opportunity to provide comments or suggest additional statements.

Subsequent Rounds: these rounds included revised versions of the statements that did not reach consensus in the previous round. Each statement was presented alongside the original version and its results (Likert scale percentages and a summary of participant comments). This iterative process continued for up to three rounds. Only the second round could include new statements, if they were suggested during the first round [10, 14, 15].

#### Data analysis

Consensus was defined as being achieved when more than 75% of participants either agreed (Likert scale score of 1 or 2) or disagreed (Likert scale score of 4 or 5) with a statement. Surveys that were less than 90% complete were excluded from the analysis. Descriptive statistical analysis was performed after each round to assess central tendency. Given that the Likert scale generates ordinal data, results were reported using the median and interquartile range [14, 16].

## Results

### Phase 1

Two focus group discussions were held, involving a total of three physiotherapists and one physiotherapist in training, two occupational therapists, and four nurses. All participants except for the physiotherapist in training, were female and had at least one year of experience working in inpatient geriatric rehabilitation with older adults who are temporarily restricted from bearing weight on a lower extremity (Table 1).

**Table 1:** Demographics focus group discussions

		1st focus group *N* (%)	2nd focus group *N* (%)
Profession	Physiotherapist	1 (25%)	2 and 1 physiotherapist in training (50%)
	Occupational therapist	1 (25%)	1 (16.67)
	Nurse	2 (50%)	2 (33.33%)
Gender	Female %	4 (100%)	5 (83.33%)
Years working at GR center	0–5 years	1 (25%)	4 (66.67%)
	5–10 years	2 (50%)	2 (33.33%)
	> 10 years	1 (25%)	

After thematically analyzing the transcripts of the focus group discussions, 80 sub-themes and seven overarching themes were identified. These were supplemented by 35 additional sub-themes and one theme derived from interviews with NWB patients and caregivers [9]. No further (sub-)themes were identified from the literature. In total, the 115 sub-themes were formatted into 115 statements, and grouped into the eight themes before being presented to the expert panel in Phase 2 (Table 2).

**Table 2:** Number of statements per theme

Theme	Phase 1: Statements focus groups N	Phase 1: Statements interviews N	Phase 2: Final statements consensus study
Definition of physical activity	9		5
Purpose of physical activity	13	2	13
Frequency of physical activity	4		3
Content of physical activity	23	2	12
Informing patients and informal caregivers	5	5	2
Involving informal caregivers	7	6	3
Stimulating and motivating physical activity	19	11	7
Role of healthcare professional		9	2
Total	80	35	47

### Phase 2

Eight researchers with a background in para(medical) professions participated in the expert panel. Of these, seven started the questionnaire, and six completed it. A total of six experts, one of whom did not complete the questionnaire, participated in the online discussion. This discussion resulted in the removal of six statements, as their mean score on the Likert scale indicated irrelevance (mean score below 1.5 on a 0–4 scale). Additionally, four criteria for the exclusion of statements were established:

1. The statement does not contribute to answering the research question.
2. The statement is similar to or overlapping with another statement.
3. The statement describes a practice that is already commonly implemented in the Netherlands.
4. Very specific statements should be reformulated into broader, more general statements, which may result in overlap with existing statements.

Following discussions with the expert panel and research team, a total of 47 statements were deemed relevant for inclusion in the consensus study (Table 2).

### Phase 3

#### Participants characteristics

In total, 76 healthcare professionals from two hospitals and 15 inpatient geriatric rehabilitation centers participated in the first round of the consensus study. Most participants were physiotherapists, followed by nurses, occupational therapists, and surgeons (Table 3). All participants provided informed consent at the start of the Delphi process. Consent was a prerequisite for participation.

**Table 3:** Characteristics of healthcare professionals participating in the Delphi study

Characteristics	Sub-category	Round 1 *N* = 76	Round 2 *N* = 51
Mean age, years (SD)		41.5 (12.1)	42 (11.7)
Mean year working experience, year (SD)		15.2 (11.0)	15.4 (10.9)
Profession in healthcare, n (%)	Physiotherapist	39 (51.3%)	28 (54.9%)
	Occupational therapist	12 (15.8%)	5 (9.8%)
	Nurses	18 (23.7%)	13 (25.5%)
	Orthopedic and trauma surgeons	7 (9.2%)	5 (9.8%)

### Round 1 of the Delphi process

**Fig. 1:**
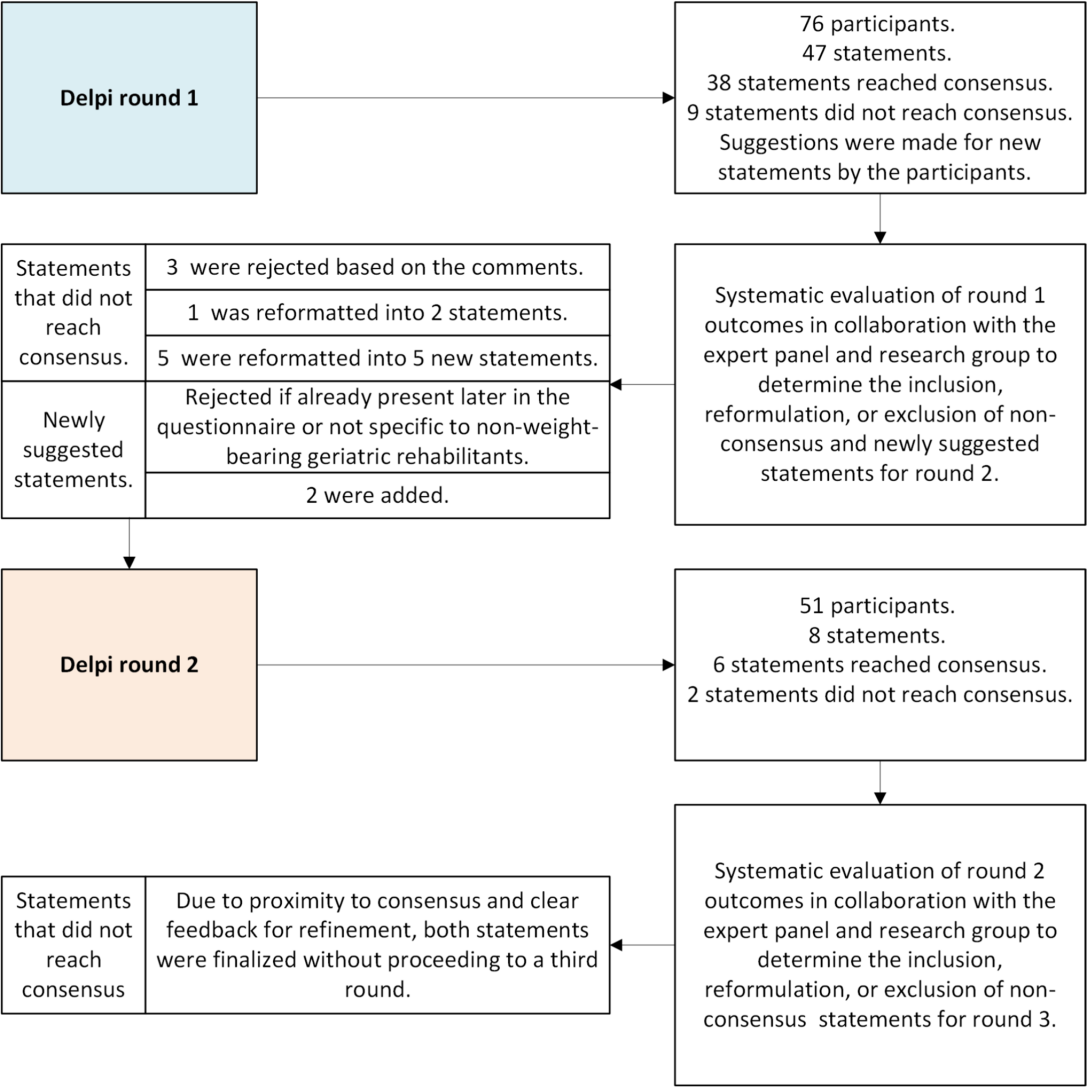
Process diagram of statement evaluation round 1 and round 2

All 76 participants completed the first round of the Delphi study, which consisted of 47 statements. Of these statements, 38 reached consensus: participants agreed on 37 statements and disagreed on one (Table 4). The nine statements that did not reach consensus, along with newly suggested statements from participants, were evaluated for the second round by the expert panel and research team. Of the statements that did not reach consensus, three were rejected based on participant comments, one was reformulated into two new statements, and five were revised. Suggestions for new statements were excluded if they were already present in the first round questionnaire or if they were not specific to NWB patients. Two suggested statements were deemed relevant and included in the second round (Fig. 1).

**Table 4:** Final list of statements regarding physical activity for older adults during the non-weight-bearing rehabilitation

Statement and round in which consensus was reached (R)	Median (IQR)	Level of agreement (%)
Definition of physical activity		
Independent mobility without bearing weight on the affected leg (e.g., using a wheelchair or walker). (R1)	1 (1)	97.4%
Performing transfers independently or with assistance from a therapist, without weight-bearing. (R1)	1 (1)	93.4%
Performing physiotherapeutic exercises as described in physical activity guidelines (strength, endurance, flexibility, balance, and bone-strengthening). (R1)	1 (1)	97.4%
Independently performing activities of daily living (ADLs). (R1)	1 (1)	92.1%
Gradually increasing sitting duration. (R1)	2 (1)	88.2%
Purpose of physical activity		
Goals are individualized and must be determined in consultation with the patient. (R1)	1 (1)	97.4%
Goals should be regularly evaluated and adjusted in collaboration with the patient. (R1)	2 (1)	94.7%
The possibility of returning home during the non-weight-bearing period should be assessed periodically. (R1)	1 (1)	96.1%
Minimize physical deconditioning so the patient can engage in meaningful activities during the non-weight-bearing period. (R1)	1 (1)	98.7%
Minimize physical deconditioning to enable the patient to resume meaningful activities as soon as possible after the non-weight-bearing period. (R1)	1 (0)	100%
Create conditions that allow the patient to return home during the non-weight-bearing period. (R1)	2 (1)	88.2%
Increase independence. (R1)	1 (1)	98.7%
Prevent comorbidities. (R1)	1 (1)	94.7%
Improving emotional state. (R1)	2 (1)	93.4%
Stimulating cognitive function. (R1)	2 (1)	84.2%
Maintaining social connections can be a secondary goal of physical activity for the patient. (R2*)	–	–
Restoring trust in one’s body. (R1)	1 (1)	100%
Frequency of physical activity		
The frequency of physical activity is individualized and should be tailored in collaboration with the patient, based on their capacity and what is needed to achieve rehabilitation goals. (R2)	2 (1)	98.0%
The non-weight-bearing patient is expected to engage in patient-regulated physical activity in addition to scheduled therapy sessions. (R1)	1 (1)	98.7%
The frequency of physical activity should be evaluated at least every two weeks to ensure it remains appropriate for the patient. (R2*)	-	-
Content of physical activity		
Based on individual goals, a physical activity plan is developed collaboratively by the therapist and the patient. (R1)	2 (1)	94.7%
When monitoring physical activity, eHealth can be used, taking into account the patient’s level of digital literacy. (R2)* Examples of eHealth tools include: motion sensors, video calling, and training apps*	2 (1)	86.3%
When training physical activity, eHealth can be used, considering the patient’s digital skills. (R2)* Examples of eHealth tools include: motion sensors, video calling, and training apps*	2 (1)	88.2%
Exercises should be provided that the non-weight-bearing patient can perform independently. (R1)	1 (1)	100%
Visual support is important when providing self-regulated exercises (e.g., videos, printed materials with photos). (R1)	2 (1)	89.5%
Incorporates resistance training. (R1)	1 (1)	98.7%
Incorporates aerobic training. (R1)	1 (1)	97.4%
Incorporates flexibility exercises aimed at preventing or reducing muscle shortening and joint limitations. (R2)	2 (1)	90.2%
Incorporates balance exercises. (R1)	2 (1)	78.9%
Incorporates bone-strengthening exercises. (R1)	2 (2)	75.0%
Includes a mix of high- and low-intensity activities. (R1)	2 (1)	89.5%
In principle, includes a combination of individual and group therapy. (R2)	2 (1)	76.5%
Informing Patients and Informal Caregivers		
The patient receives verbal and written/digital information about the benefits and risks of (in)activity during the non-weight-bearing period. (R1)	2 (1)	94.7%
Informal caregivers should be informed about the benefits and risks of (in)activity during the non-weight-bearing period. (R1)	2 (1)	78.9%
Involving informal caregivers		
Informal caregivers are asked how they wish to be involved in the rehabilitation process. If possible, this is incorporated into the rehabilitation plan. (R1)	2 (1)	76.3%
Informal caregivers are involved later in the process: during the transition to home and physical activity at home. (R1)	4 (0)	76.3% disagreed
Stimulating and motivating physical activity		
Use of eHealth. (R1)	2 (2)	75.0%
Raising the patient’s awareness of their physical capabilities. (R1)	1 (1)	100%
Involving informal caregivers. (R1)	2 (1)	89.5%
Timing pain management to facilitate physical activity. (R1)	2 (1)	86.8%
Providing reminders (e.g., setting alarms) to prompt physical activity. (R1)	2 (1)	78.9%
Making progress visible. (R1)	1.5 (1)	96.1%
Making physical activity more enjoyable, e.g., by adding a social or game element, or offering outdoor activities. (R1)	2 (1)	90.8%
Role of healthcare professionals		
All healthcare professionals within the geriatric rehabilitation centers contribute to promoting physical activity according to their role. (R1)	1 (1)	89.5%
All healthcare professionals within the geriatric rehabilitation centers are well-informed about the capabilities and limitations of patients regarding physical activity. (R1)	2 (1)	82.9%
All healthcare professionals should possess basic knowledge of physical activity and recovery. (R2)	1 (1)	96.1%

^*^Statements that did not reach consensus in the second round but were modified based on the participants’ comments and expert feedback

### Round 2 of the Delphi process

The second round of the Delphi study consisted of eight statements and was completed by 51 participants. Of these, six reached consensus (Table 4). Following discussions with the expert panel and the research team, the two statements that did not reach consensus were revised, based on participant comments and expert feedback (Table 4). As both statements were close to reaching consensus and the feedback provided clear direction for refinement, the expert panel and research team decided not to present them in a third round to the healthcare professionals (Fig. 1). An additional round was considered unnecessary and would have placed further demands on participants’ time.

### Results per theme

1. Definition of physical activity
  Consensus was reached on all statements regarding the definition of physical activity. However, based on participant comments, whether an activity is perceived as physically active depends on the patient’s physical condition upon admission to the GR center and the intensity of the activity. For example, in the context of activities of daily living, brushing one’s teeth may not be considered physically active, whereas washing oneself might be. Additional comments emphasized the importance of physical activity for maintaining independence and the need to perform such activities safely.
2. Purpose of physical activity
  Consensus was reached on 10 out of 13 statements in the first round of the Delphi study. All participants agreed that one of the key purposes of physical activity during the NWB period is to minimize physical deconditioning and to restore trust in one’s body. It was noted that maintaining or improving physical activity during this period may accelerate recovery once weight-bearing is permitted. Participants also agreed that patients should be involved in goal-setting and evaluation. However, they emphasized the importance of considering the patient’s cognitive abilities and the therapist’s expertise when determining what is appropriate and necessary.
  No consensus was reached regarding the patients’ participation in multi-disciplinary team meetings and their rehabilitation plan. Most participants commented that the plan should be discussed with the patient and that the multi-disciplinary meeting should be addressed beforehand, but that the patient should not take part in the meeting itself. As this statement was not specific to NWB rehabilitation, it was decided during the expert meeting that it would not proceed to the second round.
  For several statements, participants noted that while they agreed in principle, feasibility varied depending on the individual. This applied for example to the statement “Creating conditions that allow the patient to return home during the NWB period.”, which was considered not feasible for every person or home situation. The same applied to: “Establishing social connections.”, which did not reach consensus. After the second round it nearly reached consensus (74.5% agreed). Participants commented that while it may be a goal, it should not be considered a primary goal. Based on these comments, and in agreement with the expert panel, the statement was revised to: “Maintaining social connections can be a secondary goal of physical activity for the patient.”
3. Frequency of physical activity
  Almost all participants agreed that the patients should perform patient-regulated exercises daily outside of scheduled therapy sessions. However, the other two statements did not reach consensus in the first round. Healthcare professionals did not agree that the frequency should be tailored to the patient’s preferences. Participants commented that patients might desire more than they can physically manage or more than the GR center can realistically provide. Conversely, they might prefer less activity than is necessary to achieve their rehabilitation goals. After revision 98% of the participants agreed on this statement.
  The other statement that did not reach consensus stated that the frequency of physical activity should align with the ACSM guidelines. The participants commented that while they agreed that it should align with the ACSM guidelines, it is not feasible for NWB patients. As the statement was considered unrealistic in practice, it was not included in the second round. The statement “The frequency of physical activity should be evaluated every two weeks to ensure it remains appropriate for the patient. This can be supported by the use of eHealth tools.” was newly added to the second round but did not reach consensus. Some participants felt that evaluating every two weeks was either too frequent or not frequent enough, while others considered eHealth an unsuitable method for evaluating frequency. Based on these comments, and in agreement with the expert panel, the statement was revised after the second round to: “The frequency of physical activity should be evaluated at least every two weeks to ensure it remains appropriate for the patient.”.
4. Content of physical activity
  Consensus was reached on most statements in round one, with all participants agreeing on the importance of providing patients with patient-regulated exercises (homework). These exercises could be offered in various formats, such as paper-based instructions, pictures, or videos. There was no consensus on the use of eHealth in the first round. Healthcare professionals indicated that either they or the patient lacked sufficient knowledge to effectively use eHealth tools. Based on this feedback, the statement was revised into two separate statements for the second round, one focusing on monitoring and the other on training, while also providing examples of eHealth devices. Both revised statements reached consensus in the second round.
  Almost all healthcare professionals agreed that physical activity interventions should include resistance and aerobic exercises, as well as a combination of high- and low-intensity activities to prevent overuse injuries. Just over 75% of the participants agreed that the intervention should include balance and bone-strengthening exercises, if feasible. In the second round, most participants agreed that flexibility exercises aimed at preventing or reducing muscle shortening and joint limitations should also be included. One statement, regarding permissive weight-bearing, did not reach consensus and was excluded from the second round. It was noted that such a shift must first be initiated by surgeons before it can be incorporated into a physical activity intervention.
5. Informing patients and informal caregivers
  Consensus was reached on both informing the patient and their informal caregivers, with a higher level of agreement on informing the patient. According to participants, whether informal caregivers should be informed depends on the patient’s cognitive abilities and their personal preference regarding caregiver involvement.
6. Involving informal caregivers
  In the first round, consensus was reached that informal caregivers should be asked how they want to be involved in the rehabilitation process, and that their involvement should not be limited to the later stages, such as the transition home. A statement proposing to involve informal caregivers when rehabilitation progress stagnates, did not reach consensus. Participants noted that involvement in such cases depends on the reason for stagnation and emphasized the importance of early engagement. Given the contradiction with the statement that did reach consensus—asking caregivers about their preferred level of involvement—the expert panel decided to exclude this statement from the second round.
7. Stimulating and motivating physical activity
  Consensus was reached on all statements related to stimulating and motivating physical activity. The use of eHealth tools narrowly reached consensus, with participants noting that their effectiveness depends on the patient’s digital skills. Full consensus (100%) was achieved on the importance of raising the patient’s awareness of their physical capabilities. Making progress visible also received strong agreement (96.1%). Comments indicated that motivational techniques should be tailored to the patient’s cognitive abilities and personal preferences. However, it was also emphasized that engaging in physical activity is ultimately the patient’s own responsibility.
8. Role of healthcare professionals
  Most respondents agreed that all healthcare professionals within the rehabilitation setting contribute to promoting physical activity, each in their own way and according to their role. There was also consensus that healthcare professionals should be well-informed about the physical capabilities and limitations of patients although it was acknowledged that this is not always the case in practice. A new statement was proposed in the first round, suggesting that all healthcare professionals should possess basic knowledge of physical activity and recovery. Following discussion and refinement by the expert panel, this statement was included and reached consensus in the second round.

## Discussion

This three-phase iterative design facilitated a comprehensive exploration of healthcare professionals’ perspectives on physical activity for older adults during the NWB rehabilitation and aimed to reach consensus among a broad group of healthcare professionals on these perspectives. This process resulted in 47 statements, organized into eight themes: definition, purpose, frequency and content of physical activity, informing patients and informal caregivers, involving informal caregivers, stimulating and motivating physical activity, and the role of the healthcare professional.

We aimed to include 20 healthcare professionals from each discipline. However, the response rates varied considerably across professions. Physiotherapists were particularly responsive, comprising 51.3% (*n* = 39) of the participants. In contrast, recruiting occupational therapists and surgeons proved more challenging. This distribution appears to reflect real-world practice. As highlighted in a previous interview study with NWB patients, physiotherapists are most involved in physical activity, whereas surgeons and occupational therapists are less involved [9]. Greater involvement in physical activity may contribute to increased motivation to participate in related research [17].

During the focus group discussions, a wide range of perspectives and ideas emerged regarding physical activity during the NWB period. One such perspective was that strict non-weight-bearing should be abandoned in favor of permissive weight-bearing, which is a protocol that allows immediate weight-bearing based on pain tolerance [18]. Permissive weight-bearing has shown promising results in activities of daily living and quality of life compared to strict NWB protocols [18–20]. However, the current evidence remains limited for various types of fractures, and the international AO-guideline still recommends six weeks of non-weight-bearing [21, 22]. The statement proposing a shift from strict non-weight-bearing to permissive weight-bearing did not reach consensus. Several participants noted that therapists follow the advice given by surgeons, indicating that any transition toward permissive weight-bearing must first be initiated within the hospital setting before it can be adopted in rehabilitation centers.

Even though participants agreed with most of the statements, they noted that not all statements may be applicable to every patient. This aligns with findings from a previous consensus study among healthcare providers on optimal care for older patients with weight-bearing restrictions, which emphasized the importance of personalized physical activity [7]. In fact, multiple studies have shown that physical activity training should be tailored to the end user [6, 23, 24]. While the scope of the study by Aloraibi et al. (2021) was broader and less specifically focused on physical activity, several other similarities apart from personalized physical activity can be identified. Both studies emphasize the inclusion of strength, aerobic, and flexibility training, and both studies identify the prevention of physical deconditioning and comorbidities as objectives of physical activity. Importantly, no contradictions were identified between the statements formulated in the present study and those reported by Aloraibi et al. (2021).

The statement suggesting that the frequency of physical activity for NWB patients should align with the physical activity guideline—150–300 min of moderate or 75–150 min of vigorous physical activity per week, or a combination, plus strength training at least twice per week—did not reach consensus [25]. Participants view this recommendation as ideal but unrealistic for most NWB patients. However, when tailored to the individual and adjusted to match their perceived effort relative to their level of fitness, meeting these guidelines may be feasible [6, 25].

### Strengths and limitations

A strength of this study was the participation of healthcare professionals from 17 different facilities, including two hospitals and 15 GR centers. Although each GR center and hospital often operates in its own way, a high level of consensus was reached on most statements, enhancing the relevance and generalizability of the results within the Dutch context. However, as this study was conducted in the Netherlands, differences in healthcare systems and cultural values may limit the transferability of the results to other countries. The involvement of the expert panel strengthened the study by reducing the risk of research bias during the selection and formulation of relevant statements for inclusion in the consensus study in the first and subsequent rounds. Since surgeons are typically not involved in facilitating physical activity but are responsible for prescribing the NWB restrictions, we sought to collect their opinions on the statements without including them in the initial information-gathering phase. Therefore, they were not invited to participate in the focus group discussions but were included in the Delphi study. A potential limitation is that this may have led to missing relevant input. However, surgeons were given the opportunity to provide feedback through the comment section in the Delphi questionnaire. A limitation is that only seven surgeons were included. Several hospitals declined participation due to survey fatigue, indicating that questionnaires longer than 5–10 min were not feasible for their staff.

## Conclusion and recommendations

This study provides a consensus-based foundation for developing physical activity interventions for older patients during the non-weight-bearing period. While there was broad agreement on most statements, participants emphasized the importance of tailoring interventions to the patient’s individual condition, abilities, and goals.

Physical activity should aim to minimize physical deconditioning and restore trust in one’s body, combining scheduled therapy with self-regulated exercises. The content of the intervention should be goal-oriented and include different exercise types, such as strength and aerobic components. Although healthcare professionals remain cautious, there was agreement that eHealth tools can support monitoring, training, and motivating physical activity, provided they match the digital skills of the patient.

Patients and informal caregivers should be informed about physical activity during the non-weight-bearing period, and informal caregivers should be involved from the start of rehabilitation, based on their preferences. Raising awareness of physical capabilities and making progress visible were identified as key motivators, although physical activity also remains the patient’s own responsibility. All healthcare professionals play a role in promoting physical activity and should possess basic knowledge of physical activity and recovery.

## Supplementary Information

Below is the link to the electronic supplementary material.

Supplementary file1 (DOCX 23 KB)

## Data Availability

The datasets used and/or analyzed during the current study are available from the corresponding author on reasonable request.
