# Supplementary material for: Healthcare professionals consensus on purpose, content and frequency of physical activity for older adults during the non-weight-bearing rehabilitation: an iterative consensus study
Source: Eur Geriatr Med. 2026 Apr 12;17(3):1545–56. doi: 10.1007/s41999-026-01463-5 (PMC13309379; doi:10.1007/s41999-026-01463-5)
Supplement: Supplementary file 1 — Supplementary file1 (DOCX 23 KB) [file 41999_2026_1463_MOESM1_ESM.docx]

**Supporting information**

Table 5: Statements from the first Delphi round

| Statement | Median (IQR) | Level of agreement (%) |
| --- | --- | --- |
| Definition of Physical Activity | |  |
| 1. Independent mobility without bearing weight on the affected leg (e.g., using a wheelchair or walker). | 1 (1) | 97.4% |
| 2. Performing transfers independently or with assistance from a therapist, without weight-bearing. | 1 (1) | 93.4% |
| 3. Performing physiotherapeutic exercises as described in physical activity guidelines (strength, endurance, flexibility, balance, and bone strengthening). | 1 (1) | 97.4% |
| 4. Independently performing activities of daily living (ADLs). | 1 (1) | 92.1% |
| 5. Gradually increasing sitting duration. | 2 (1) | 88.2% |
| Purpose of Physical Activity | |  |
| 6. Goals are individualized and must be determined in consultation with the patient. | 1 (1) | 97.4% |
| 7. Goals should be regularly evaluated and adjusted in collaboration with the patient. | 2 (1) | 94.7% |
| 8. The patient is an active participant in the multidisciplinary team meetings and is directly involved in the rehabilitation plan. | 3 (2) | 31.6%* |
| 9. The possibility of returning home during the non-weight-bearing period should be assessed periodic. | 1 (1) | 96.1% |
| 10. Minimize physical deconditioning so the patient can engage in meaningful activities during the non-weight-bearing period. | 1 (1) | 98.7% |
| 11. Minimize physical deconditioning to enable the patient to resume meaningful activities as soon as possible after the non-weight-bearing period. | 1 (0) | 100% |
| 12. Create conditions that allow the patient to return home during the non-weight-bearing period. | 2 (1) | 88.2% |
| 13. Increase independence. | 1 (1) | 98.7% |
| 14. Prevent comorbidities. | 1 (1) | 94.7% |
| 15. Improving emotional state. | 2 (1) | 93.4% |
| 16. Stimulating cognitive function. | 2 (1) | 84.2% |
| 17. Establishing social connections. | 2 (1) | 53.9%* |
| 18. Restoring trust in one’s body. | 1 (1) | 100% |
| Frequency of Physical Activity | |  |
| 19. Frequency is individualized and should be tailored to the patient’s capacity and preferences. | 2 (2) | 71.1%* |
| 20. Frequency should align with ACSM guidelines: 150–300 minutes of moderate or 75–150 minutes of vigorous physical activity per week, or a combination, plus strength training at least twice per week. | 2.5 (1) | 50.0% * |
| 21. The non-weight-bearing patient is expected to engage in patient-regulated physical activity in addition to scheduled therapy sessions. | 1 (1) | 98.7% |
| Content of Physical Activity | |  |
| 22. Based on individual goals, a physical activity plan is developed collaboratively by the therapist and the patient. | 2 (1) | 94.7% |
| 23. eHealth can be used for both monitoring and training physical activity. | 2 (2) | 73.7%* |
| 24. Exercises should be provided that the non-weight-bearing patient can perform independently. | 1 (1) | 100% |
| 25. Visual support is important when providing self-regulated exercises (e.g., videos, printed materials with photos). | 2 (1) | 89.5% |
| 26. The policy of strict non-weight-bearing should be abandoned in favor of permissive weight-bearing, a protocol allowing immediately weight-bearing based on pain tolerance. | 3 (1) | 43.4%* |
| 27. Incorporates resistance training. | 1 (1) | 98.7% |
| 28. Incorporates aerobic training. | 1 (1) | 97.4% |
| 29. Incorporates flexibility exercises. | 2 (2) | 69.7%* |
| 30. Incorporates balance exercises. | 2 (1) | 78.9% |
| 31. Incorporates bone-strengthening exercises. | 2 (2) | 75.0% |
| 32. Includes a mix of high- and low-intensity activities. | 2 (1) | 89.5% |
| 33. Includes a combination of individual and group therapy. | 2 (2) | 61.8%* |
| Informing Patients and Informal Caregivers | |  |
| 34. The patient receives verbal and written/digital information about the benefits and risks of (in)activity during the non-weight-bearing period. | 2 (1) | 94.7% |
| 35. Informal caregivers should be informed about the benefits and risks of (in)activity during the non-weight-bearing period. | 2 (1) | 78.9% |
| Involving Informal Caregivers | |  |
| 36. Informal caregivers are asked how they wish to be involved in the rehabilitation process. If possible, this is incorporated into the rehabilitation plan. | 2 (1) | 76.3% |
| 37. Informal caregivers are involved in the rehabilitation process during the non-weight-bearing period if progress stagnates. | 2 (1) | 72.4%* |
| 38. Informal caregivers are involved later in the process: during the transition to home and physical activity at home. | 4 (0) | 76.3% disagreed |
| Stimulating and Motivating Physical Activity | |  |
| 39. Use of eHealth. | 2 (2) | 75.0% |
| 40. Raising the patient’s awareness of their physical capabilities. | 1 (1) | 100% |
| 41. Involving informal caregivers. | 2 (1) | 89.5% |
| 42. Timing pain management to facilitate physical activity. | 2 (1) | 86.8% |
| 43. Providing reminders (e.g., setting alarms) to prompt physical activity. | 2 (1) | 78.9% |
| 44. Making progress visible. | 1.5 (1) | 96.1% |
| 45. Making physical activity more enjoyable, e.g., by adding a social or game element, or offering outdoor activities. | 2 (1) | 90.8% |
| Role of Healthcare Professionals | |  |
| 46. All healthcare professionals within the geriatric rehabilitation centers contribute to promoting physical activity according to their role. | 1 (1) | 89.5% |
| 47. All healthcare professionals within the geriatric rehabilitation centers are well-informed about the capabilities and limitations of patients regarding physical activity. | 2 (1) | 82.9% |

* No consensus was reached

Table 6: Statements from the second Delphi round

| Statement | Median (IQR) | Level of agreement (%) |
| --- | --- | --- |
| Purpose of Physical Activity |  |  |
| 17. Maintaining social connections can be a goal of physical activity for the patient. | 2 (1) | 74.5%* |
| Frequency of Physical Activity |  |  |
| 19. The frequency of physical activity is individualized and should be tailored in collaboration with the patient, based on their capacity and what is needed to achieve rehabilitation goals. | 2 (1) | 98.0% |
| 48. The frequency of physical activity should be evaluated every two weeks to ensure it remains appropriate for the patient. This can be supported by the use of eHealth tools. | 2 (1) | 66.7%* |
| Content of Physical Activity |  |  |
| 23.1. When monitoring physical activity, eHealth can be used, taking into account the patient’s level of digital literacy.  *Examples of eHealth tools include: motion sensors, video calling, and training apps.* | 2 (1) | 86.3% |
| 23.2. When training physical activity, eHealth can be used, considering the patient’s digital skills.  *Examples of eHealth tools include: motion sensors, video calling, and training apps.* | 2 (1) | 88.2% |
| 29. Incorporates flexibility exercises aimed at preventing or reducing muscle shortening and joint limitations. | 2 (1) | 90.2% |
| 33. In principle, includes a combination of individual and group therapy. | 2 (1) | 76.5% |
| Role of Healthcare Professionals |  |  |
| 49. All healthcare professionals should possess basic knowledge of physical activity and recovery. | 1 (1) | 96.1% |

* No consensus was reached

Tabel 7: Modified statements after the second Delphi round

| Statement |
| --- |
| Purpose of Physical Activity |
| 17. Maintaining social connections can be a secondary goal of physical activity for the patient. |
| Frequency of Physical Activity |
| 48. The frequency of physical activity should be evaluated at least every two weeks to ensure it remains appropriate for the patient. |
